# Unraveling the Complexity of DNA Radiation Damage Using DNA Nanotechnology

**DOI:** 10.1021/acs.accounts.4c00121

**Published:** 2024-05-23

**Authors:** João Ameixa, Ilko Bald

**Affiliations:** † Institute of Chemistry, Hybrid Nanostructures, University of Potsdam, Karl-Liebknecht-Str. 24-25, 14476 Potsdam, Germany; ‡ Centre of Physics and Technological Research (CEFITEC), Department of Physics, NOVA School of Science and Technology, University NOVA of Lisbon, Campus de Caparica 2829-516, Portugal

## Abstract

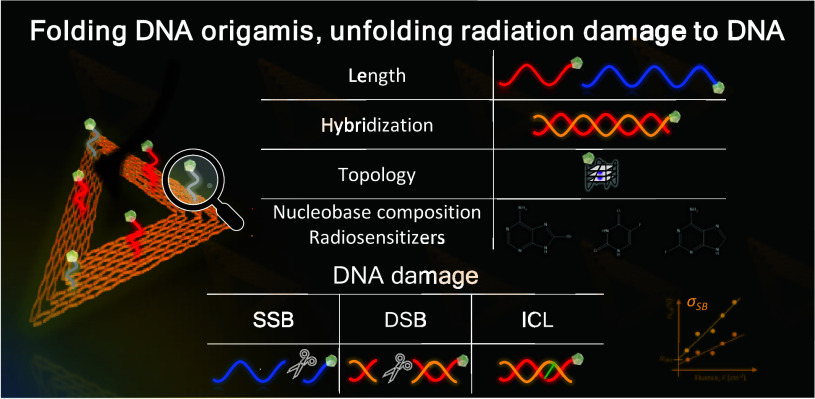

Radiation cancer therapies use different ionizing radiation qualities that damage DNA molecules in tumor cells by a yet not completely understood plethora of mechanisms and processes. While the direct action of the radiation is significant, the byproducts of the water radiolysis, mainly secondary low-energy electrons (LEEs, <20 eV) and reactive oxygen species (ROS), can also efficiently cause DNA damage, in terms of DNA strand breakage or DNA interstrand cross-linking. As a result, these types of DNA damage evolve into mutations hindering DNA replication, leading to cancer cell death. Concomitant chemo-radiotherapy explores the addition of radiosensitizing therapeutics commonly targeting DNA, such as platinum derivatives and halogenated nucleosides, to enhance the harmful effects of ionizing radiation on the DNA molecule. Further complicating the landscape of DNA damage are secondary structures such as G-quadruplexes occurring in telomeric DNA. These structures protect DNA from radiation damage, rendering them as promising targets for new and more selective cancer radiation treatments, rather than targeting linear DNA. However, despite extensive research, there is no single paradigm approach to understanding the mysterious way in which ionizing radiation causes DNA damage. This is due to the multidisciplinary nature of the field of research, which deals with multiple levels of biological organization, from the molecular building blocks of life toward cells and organisms, as well as with complex multiscale radiation-induced effects. Also, intrinsic DNA features, such as DNA topology and specific oligonucleotide sequences, strongly influence its response to damage from ionizing radiation. In this Account, we present our studies focused on the absolute quantification of photon- and low-energy electron-induced DNA damage in strategically selected target DNA sequences. Our methodology involves using DNA origami nanostructures, specifically the Rothemund triangle, as a platform to expose DNA sequences to either low-energy electrons or vacuum-ultraviolet (VUV, <15 eV) photons and subsequent atomic force microscopy (AFM) analysis. Through this approach, the effects of the DNA sequence, incorporation of halogenated radiosensitizers, DNA topology, and the radiation quality on radiation-induced DNA strand breakage have been systematically assessed and correlated with fundamental photon- and electron-driven mechanisms underlying DNA radiation damage. At lower energies, these mechanisms include dissociative electron attachment (DEA), where electrons attach to DNA molecules causing strand breaks, and dissociative photoexcitation of DNA. Additionally, further dissociative processes such as photoionization and electron impact contribute to the complex cascade of DNA damage events induced by ionizing radiation. We expect that emerging DNA origami-based approaches will lead to a paradigm shift in research fields associated with DNA damage and suggest future directions, which can foster the development of technological applications in nanomedicine, e.g., optimized cancer treatments or the molecular design of optimized radiosensitizing therapeutics.

## Key References

- WangC.; EbelK.; HeinzeK.; Resch-GengerU.; BaldI.Quantum Yield of DNA Strand Breaks under Photoexcitation of a Molecular Ruby. Chem.—Eur. J.2023, 29( (23), ), e20220371910.1002/chem.20220371936734093 .^1^*The [Cr(ddpd)*_*2*_*]*^*3+*^*complex induces DNA strand breaks under UV/vis light, making it a potential photosensitizer for photodynamic therapy. By combining DNA origami technology with atomic force microscopy, the quantum yield of strand breaks was quantified to be between 1 and 4%*.
- EbelK.; BaldI.Low-Energy (5–20 EV) Electron-Induced Single and Double Strand Breaks in Well-Defined DNA Sequences. J. Phys. Chem. Lett.2022, 13( (22), ), 4871–487610.1021/acs.jpclett.2c0068435617198 PMC9189919.^2^*This study with DNA origami nanostructures and AFM imaging introduces a novel approach allowing for the determination of absolute cross sections for electron-induced DNA double-strand breaks*.
- VogelS.; EbelK.; SchürmannR. M.; HeckC.; MeilingT.; MilosavljevicA. R.; GiulianiA.; BaldI.Vacuum-UV and Low-Energy Electron-Induced DNA Strand Breaks - Influence of the DNA Sequence and Substrate. ChemPhysChem2019, 20( (6), ), 823–83010.1002/cphc.20180115230719805 .^3^*This paper shows that the cross sections for DNA strand breakage induced by electrons are not only influenced by the nucleobase sequence but are also 1 to 2 orders of magnitude larger compared to those induced by VUV photons.*
- SchürmannR.; TseringT.; TanzerK.; DeniflS.; KumarS. V. K.; BaldI.Resonant Formation of Strand Breaks in Sensitized Oligonucleotides Induced by Low-Energy Electrons (0.5–9 EV). Angewandte Chemie - International Edition2017, 56( (36), ), 10952–1095510.1002/anie.20170550428670830 .^4^*For the first time, the effect of incorporating 8-bromoadenine, a potential radiosensitizer, into a DNA sequence on electron-induced DNA strand breakage was quantified. AFM analysis of DNA origami nanostructures yielded an average strand break enhancement factor of 1.9 ± 0.6*.

## 1 Introduction

Cancer is one of the most challenging health issues of our time.^5,6^ To kill malignant tumor cells, radiation cancer therapies make use of different ionizing radiation qualities, exploiting their ability to inflict substantial damage on the DNA within tumor cells. When radiation interacts with water, its radiolysis is triggered, generating secondary particles such as reactive oxygen species (ROS) and low-energy electrons (LEEs). Because about 80% of the cellular content is water, this is the most important initial reaction producing ROS and LEEs, while they can also result from direct ionization of biomolecules such as DNA.^7^ LEEs, with energies of less than 20 eV, can cause significant damage to DNA. Through dissociative electron attachment processes, they effectively initiate DNA strand breaks and interstrand cross-linking, setting off a cascade of events that impede DNA replication and lead to the death of cancer cells.^8−10^

To further enhance the efficacy of radiation therapies, concomitant chemo-radiotherapy has emerged as a promising approach.^11,12^ This strategy involves administering a radiosensitizing therapeutic which commonly targets DNA, such as platinum derivatives^13^ and halogenated nucleosides,^14−17^ alongside radiation. This combined approach enhances the damaging effect of ionizing radiation on DNA molecules, enhancing tumor local control.

However, the various mechanisms and processes through which ionizing radiation exactly damages DNA remain complex and yet not fully understood. To elucidate these mechanisms, an interdisciplinary approach is followed spanning multiple levels of complexity, from the study of isolated building blocks of life to entire cells and organisms. Moreover, it deals with the multiscale spatial and temporal effects of radiation exposure. Additionally, intrinsic features of DNA, including its precise nucleobase composition, conformation, hybridization, and topology, significantly change its response to ionizing radiation.

In this context, our investigations focus on precisely measuring the damage caused to specific DNA sequences by both LEEs^2,18−20^ and photons.^3,21,22^ This Account presents our recent findings, featuring an innovative experimental approach that utilizes strategically designed DNA origami nanostructures based on the Rothemund triangle,^23^ coupled with atomic force microscopy (AFM) analysis. Our research has provided invaluable insights into DNA radiation damage, highlighting effects such as nucleobase composition,^3,22^ the incorporation of halogenated nucleobases,^4,18,21^ DNA topology,^24^ hybridization,^2^ and length,^25^ all of which influence radiation-induced DNA strand breakage.

As we present our findings, we anticipate that the flourishing field of DNA nanotechnology-based solutions will promote further changes in the domain of DNA damage research. These advances hold the promise of fostering the development of transformative applications in nanomedicine, from optimized cancer treatments to the tailored design of highly targeted radiosensitizing agents.

### 1.1 Experimental Technique: DNA Nanotechnology for Determining Radiation Damage to DNA

We have developed an experimental approach that enables the precise determination of absolute cross sections for LEE-induced DNA single-^19,20^ and double-strand breaks^2^ using DNA origami nanostructures. The subsequent discussions are based on this experimental scheme, where Rothemund triangles^23^ serve as platforms exposing biotinylated oligonucleotide target sequences to radiation. DNA origami, a nanotechnological technique, involves the precise folding of a single-stranded DNA scaffold through the incorporation of staple strands, yielding customizable nanostructures. This technique is significantly interesting in DNA radiation damage research due to its capabilities for the absolute quantification of DNA strand breakage, high sensitivity, and the potential to investigate sequence dependencies, enabling the direct comparison of different sequences (e.g., sensitized and nonsensitized) within a single irradiation experiment. Following irradiation, samples undergo rinsing and incubation with streptavidin (SAv). SAv efficiently binds to biotin (Bt), and due to its dimensions (4.5 × 4.5 × 5 nm^3^), intact biotinylated DNA strands become visible as bright spots in AFM images. The total number of visible SAv spots on a DNA nanostructure corresponds to the total number of intact DNA target sequences. Conversely, the absence of an expected SAv spot indicates a single DNA strand break, as the target sequence loses the Bt label through this process.

Subsequent to AFM analysis of samples exposed to gradually higher fluence values, absolute cross sections for DNA single-strand breaks, *σ*_SSB_, can be statistically determined using the procedure described in detail by Rackwitz et al.^20^ For fluence values, *F*, below the saturation threshold, the number of single-strand breaks from the AFM analysis, *N*_SB_, follows a linear relationship with fluence, *F* (eq 1):
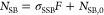
1

Here, *N*_SB,0_ represents the number of strand breaks determined from AFM analysis of a nonirradiated control sample. In the low-fluence regime, the dose–response curve is linear, meaning that the number of single-strand breaks increases linearly with fluence until saturation occurs.^19,26^

## 2 DNA Damage Induced upon VUV Irradiation (30–220 nm; 6.20–41.3 eV)

**Figure 1:**
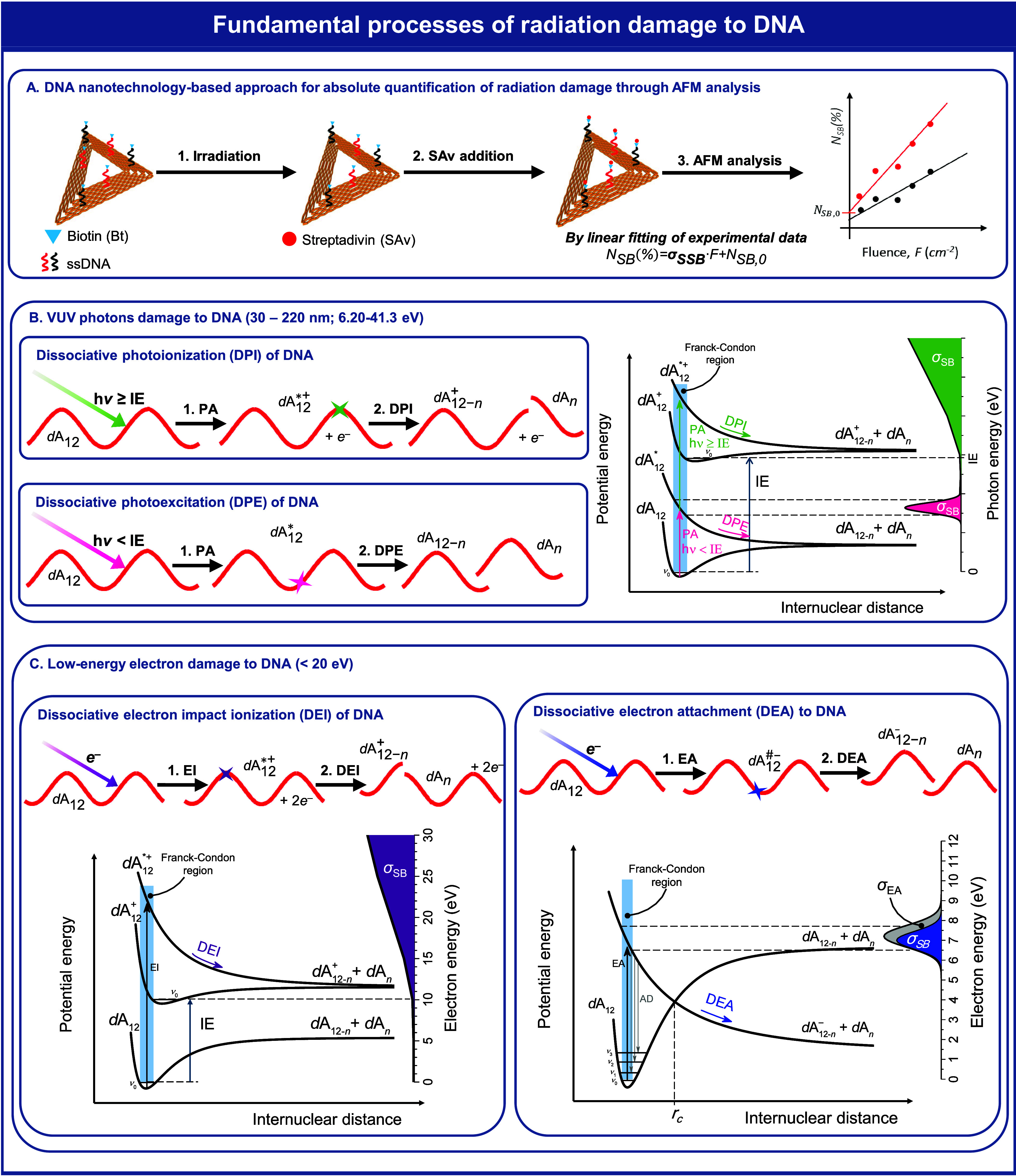
Schematic overview of our experimental approach for the absolute quantification of DNA single-strand breaks induced by electrons and photons. Additionally, it presents fundamental mechanisms underlying DNA radiation damage caused by photons and low-energy electrons. Simplified potential energy diagrams depict quasi-diatomic dissociation within the DNA molecule, dA_12_, complemented by a graph illustrating the energy-dependent absolute cross section for photon-induced or low-energy electron-induced DNA single-strand breaks, σ_SB_. Panel A outlines the experimental procedure employed for the absolute quantification of DNA single-strand breaks induced by radiation. Panels B and C showcase two fundamental mechanisms of photon and low-energy electron damage to DNA, respectively, within the target sequence dA_12_. Following DNA photoabsorption (PA), damage may arise through dissociative photoionization (DPI) and dissociative photoexcitation (DPE). Low-energy electron DNA damage may occur via dissociative electron impact ionization (DEI) or dissociative electron attachment (DEA), subsequent to electron impact (EI) or electron attachment (EA) to DNA, respectively. Below the crossing distance (*r*_c_), autodetachment (AD) of the captured electron leaves the DNA in a vibrationally excited state. In DEA to DNA, the energy dependence of the cross section for single-strand breaks, σ_SB_, overlaps with the electron attachment cross section, σ_EA_.

UV photon-induced DNA single-strand breaks (SSBs) result directly from dissociative processes, involving either the DNA backbone or the DNA nucleobases. Namely, dissociative photoexcitation occurs at photon energies below the ionization energy (IE), and dissociative photoionization (DPI) occurs at photon energies above the ionization threshold, as illustrated in Figure 1. The vertical IE of individual DNA subunits ranges from 8.2 to 11.3 eV.^27−29^ The ionization energies of DNA/RNA nucleobases are between 8.0 and 9.5 eV and follow this trend: IE(G) < IE(A) < IE(C) < IE(T) < IE(U). At or above IE, dissociative photoionization can occur, as shown by eq 2 for the DNA sequence 5′-d(A_12_), where the superscript * denotes that an A nucleobase or a subunit of the backbone is in a vibrationally or electronically excited state. At UV photon energies below the IE of individual DNA subunits, dissociative photoionization processes may induce DNA strand breakage as illustrated by eq 3.
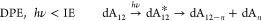
2
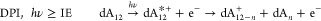
3

In 2015, Vogel et al.^22^ introduced an experimental scheme for determining absolute cross sections for UV photon-induced DNA strand breakage using DNA origami nanostructures and AFM analysis. DNA origami nanostructures deposited onto CaF_2_ (or Si) substrates were irradiated with VUV photons at the DISCO/APEX beamline of the synchrotron SOLEIL in argon (Ar) at atmospheric pressure.^3^ For a comprehensive description of the experimental setup, the reader is referred to references (21), (22), and (30). An overview of all absolute cross-section values for VUV photon-induced DNA single strand breaks determined is given in the Supporting Information (Table S1).

**Figure 2:**
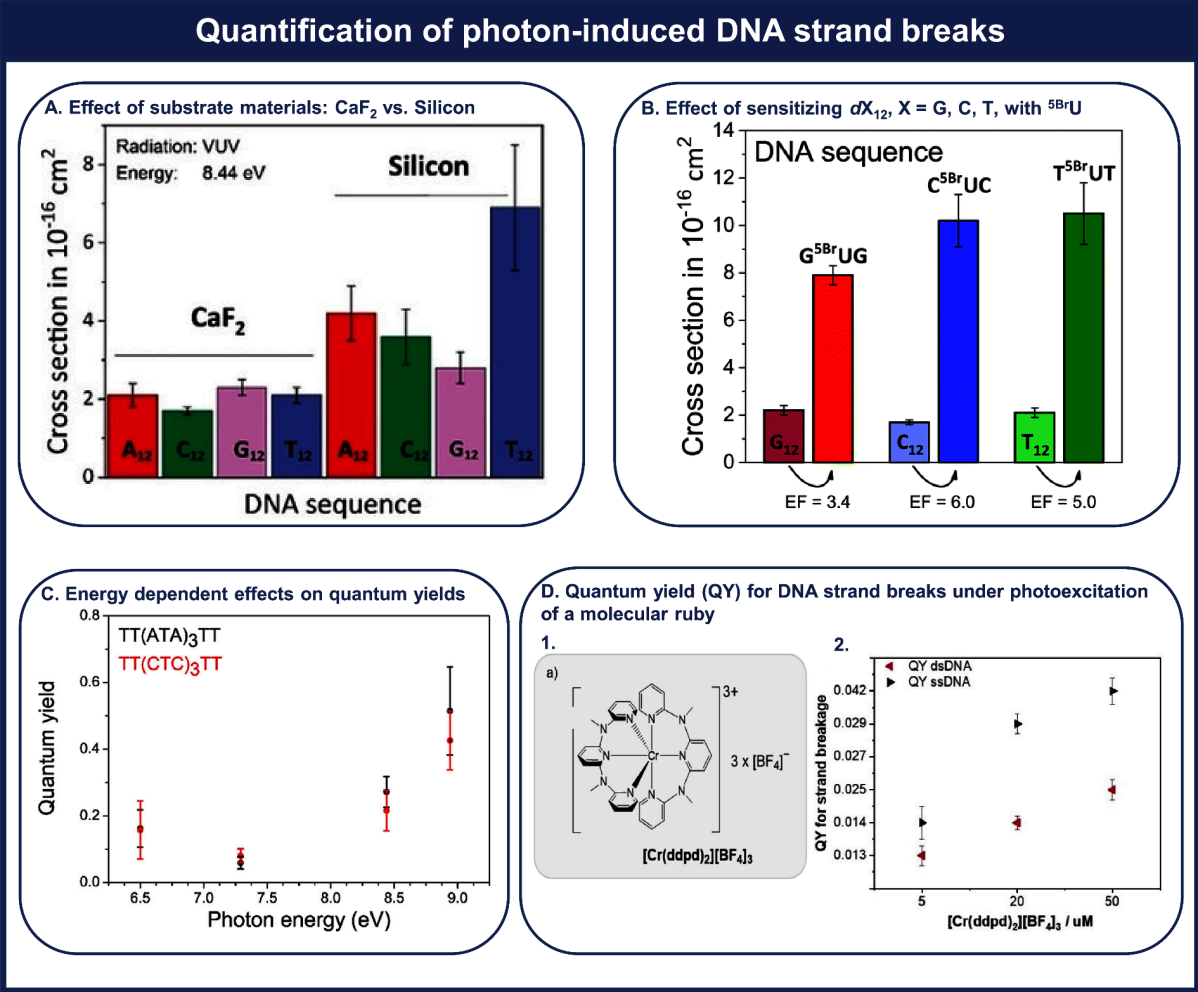
Panel A shows a comparison of the effects of two different substrates on photon-induced strand breaks in 5′-dX_12_, where X = A, C, G, T. Adapted with permission from ref (3). Copyright 2019 Wiley-VCH. Panel B depicts the effect of incorporating 5-bromouracil (^5Br^U) into target sequences 5′-dX_12_, where X = G, C, T, by a comparison between absolute cross sections for photon single-strand breakage. Adapted with permission from ref (21). Copyright 2019 PCCP Owner Societies. Panel C presents the quantum yield for single-strand breaks in the two DNA sequences, 5′-d(TT(XTX)_3_TT), where X = A, C. Adapted with permission from ref (22). Copyright 2015 American Chemical Society. Panel D shows (1) the molecular structure of the photosensitizer [Cr(ddpd)_2_][BF_4_]_3_ and (2) quantum yields for strand breakage in ss- and ds-DNA sequences. Adapted with permission from ref (1). Copyright 2023 Wiley-VCH.

Vogel et al.^21,30^ quantified the effect of secondary low-energy electrons produced within the substrate upon UV exposure on inducing DNA strand breaks. To this end, four different mononucleobase DNA sequences, 5′-d(A_12_), 5′-d(C_12_), 5′-d(G_12_), and 5′-d(T_12_), were irradiated with 8.44 eV VUV photons on CaF_2_ and Si as substrate materials. Such VUV photons produce low-energy electrons with an energy distribution peaking at 3.6 eV in Si, while CaF_2_ is a material known to be transparent to VUV radiation, in which secondary effects are not expected, such as secondary low-energy electron production or heating. In Figure 2A, it is shown that the cross section for DNA strand breakage irradiated on Si is 2 to 3 times higher than that obtained with CaF_2_ substrates and shows this trend: 5′-d(T_12_) > 5′-d(A_12_) > 5′-d(C_12_) > 5′-d(G_12_). The generated secondary low-energy electrons in Si lead to an enhancement in the observed cross sections on Si.

Vogel et al.^21^ also investigated the concurrent effects of the incorporation of two radiosensitizers—8-bromoadenine (^8Br^A), a halogenated purine, and 5-bromouracil (^5Br^U), a halogenated pyrimidine—on VUV-induced DNA strand breakage. In the study involving the radiosensitizer ^5Br^U, Vogel et al.^21^ have demonstrated that VUV photons can efficiently induce SSBs in the DNA sequences 5′-d(TT(X^5Br^UX)_3_TT) deposited onto CaF_2_. Figure 2B shows that the absolute-cross cross sections for SSBs induced by 8.44 eV VUV photons are clearly sequence-dependent as reflected in the enhancement factor (EF) trend: G^5Br^UG (EF = 3.4) < T^5Br^UT (EF = 5.0) < C^5Br^UC (EF = 6.0).

Based on the absolute cross sections for DNA single-strand breaks, our approach allows the determination of quantum yields (QY) for photon-induced DNA damage. This ratio reflects the correlation between DNA strand breaks and the number of absorbed photons. Vogel et al.^22^ conducted a study on energy-dependent quantum yields for strand breaks in the target sequences 5′-d(TT(XTX)_3_TT), X = A, C. The determined QYs, as presented in Figure 2C, range from a minimum of 6–8% at 7.29 eV to up to 16% at 6.5 eV and 40–50% at 8.94 eV for both sequences. This demonstrates the clearly high efficiency of UV photons in causing DNA strand breaks.

Recently, Wang et al.^1^ assessed the effectiveness of the water-soluble transition-metal complex [Cr(ddpd)_2_]^3+^ (ddpd = *N*,*N*′-dimethyl-*N*,*N*′-dipyridine-2-ylpyridine-2,6-diamine), as illustrated in Figure 2D.1, in inducing DNA damage upon exposure to UV/vis radiation. As shown in Figure 2D.2, they determined the QY for DNA single- and double-strand breaks under UVA excitation of the proposed photosensitizer to be in the range of 1–4%. As the strand breaks are a result of intermediate formation of triplet oxygen, this ratio suggests that [Cr(ddpd)_2_]^3+^ effectively converts photons into DNA strand breaks.

## 3 DNA Damage Induced by Low-Energy Electrons (<20 eV)

This subsection provides an overview of DNA damage caused by low-energy electrons (LEEs), with a focus on observations using AFM imaging of DNA origami nanostructures. All measured absolute cross section values for electron-induced DNA strand breakage are given in the Supporting Information (Tables S2 and S3).

By Monte Carlo track simulations and experiments,^31−33^ it has been shown that the radiolysis of water with fast protons and helium ions (MeV) yields secondary electrons (SEs) with a kinetic energy distribution below 100 eV and with a maximum at about 9–10 eV. SEs with energy below 20 eV are referred to as low-energy electrons (LEEs).

Following SE formation, these lose energy through ionization and vibrational excitation events, leading to the formation of electrons in various stages of solvation.^9,10^ These include quasi-free electrons (*e*_qf_^–^), prehydrated electrons (*e*_pre_^–^, and fully solvated electrons (*e*_aq_^–^). The reactivity of SEs varies with electron energy, solvation state, and distance to other reactive species.^9,10,34^ On a femtosecond time scale, DNA or its subunits can capture *e*_qf_^–^, forming temporary negative ions (TNIs). TNIs can decay through dissociative electron attachment (DEA) processes,^35,36^ resulting in the production of negatively charged ions and neutral radicals. For example, eq 4 illustrates the DEA process in the DNA sequence 5′-d(A_12_), leading to strand breakage, in which the superscript ^#^ indicates that an A nucleobase or a subunit of the backbone is in a metastable transient state. Alternatively, TNIs may decay by spontaneously emitting the extra electron, yielding the precursor neutral molecule in an excited state.^37^ The mechanisms underlying DNA single-strand breaks induced by LEEs have been extensively studied both experimentally and theoretically, with details available in previous research papers.^8−10,38−42^ Notably, Schürmann et al.^43^ offer a comprehensive comparison of various experimental studies conducted under different degrees of complexity and conditions. Figure 1 schematically provides simplified molecular mechanisms underlying electron-induced DNA strand breaks, along with respective energy-dependent absolute cross sections. At higher electron energies above the ionization threshold of DNA, other threshold processes such as dissociative electron ionization (DI) can also cause the fragmentation of DNA subunits into a cationic fragment and a radical fragment. For instance, eq 5 describes the DI process potentially leading to strand breaks in the DNA sequence 5′-d(A_12_). At electron energies below the ionization threshold, other electron-induced reactions, such as neutral dissociation (ND) and dipolar dissociation (DD), may initiate DNA damage. These threshold processes involve the direct scattering of an incoming electron of energy ε_1_ by a DNA subunit, leading to the formation of a transient neutral excited species that subsequently decays through bond dissociation to yield two neutral fragments, or a cation–anion pair. ε_2_ is the inelastically scattered electron’s energy. Although it is yet to be demonstrated whether such processes may also initiate electron-induced DNA cleavage, eqs 6 and 7 describe the ND and DD processes causing a hypothetical strand break in 5′-d(A_12_).
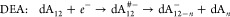
4
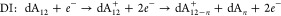
5
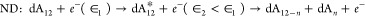
6
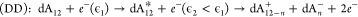
7

**Figure 3:**
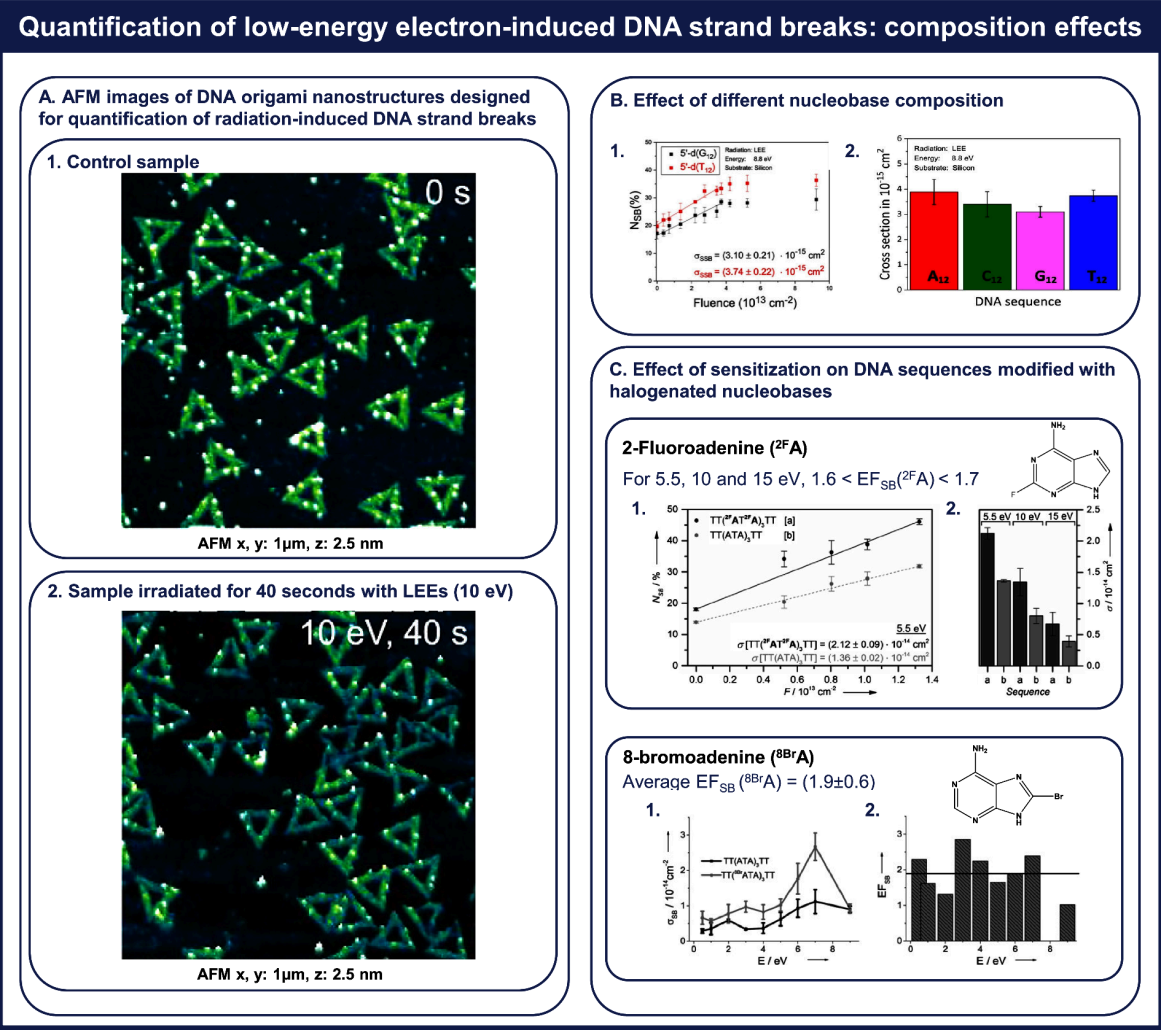
Determination of enhancement factors by direct comparison within a single irradiation experiment. Panel A showcases the impact of LEE irradiation in AFM images of (1) a nonirradiated control sample and (2) a sample exposed for 40 s to 10 eV LEEs. Adapted with permission from ref (20). Copyright 2017 Springer Nature. Panel B illustrates the effects of nucleobase composition, showing (1) the relative number of strand breaks in 5′-d(G_12_) and 5′-d(T_12_) as a function of fluence and (2) a comparison between the determined absolute cross sections for electron-induced cross sections for single strand breaks in 5′-d(X_12_), X = A, C, G, T. Adapted with permission from ref (3). Copyright 2019 Wiley-VCH. Panel C showcases the effects of modifying DNA sequences with 2-fluoroadenine (^2F^A) or 8-bromoadenine (^8Br^A) on electron-induced strand breaks. For the incorporation of ^2F^A, it displays (1) the number of relative strand single breaks in 5′-d(TT(^2F^A T^2F^A)_3_TT) (sequence a) and 5′-d(TT(ATA)_3_TT) (sequence b) and (2) a comparison between the determined absolute cross sections for electron-induced single strand breaks in sequences a and b. Additionally, it provides the average enhancement factor for single strand breakage due to the incorporation of two ^2F^A nucleobases, denoted as EF_SB_(^2F^A). Furthermore, for the incorporation of ^8Br^A, it is presented as (1) determined absolute cross sections for electron-induced single strand breaks in 5′-d(TT(XTA)_3_TT), where X = A, ^8Br^A and (2) a comparison between the obtained EF_SB_ as a function of the electron energy and the average EF_SB_(^8Br^A). Adapted with permission from refs (4) and (18). Copyrights 2016 and 2017 Wiley-VCH.

Rackwitz et al.^20^ have previously described in detail our experimental setup employed for LEE irradiation under high-vacuum conditions. The impact of this irradiation is evident in the AFM images of DNA origami nanostructures as shown in Figure 3A, representing (1) a control, nonirradiated sample and (2) a sample exposed to 10 eV LEEs for 40 s.

### 3.1 Effects of Nucleobase Composition on Low-Energy Electron (LEE)-Induced DNA Damage

Vogel et al.^21^ conducted studies to investigate how the composition of nucleobases affects LEE-induced DNA strand breaks by irradiating four different DNA sequences, 5′-d(X_12_), where X = A, C, T, G at a specific electron energy of 8.8 eV. Figure 3B illustrates the number of single-strand breaks in 5′-d(G_12_) and 5′-d(T_12_) as a function of the electron fluence. The data, as listed in Tables S2 and S3, indicate that the highest cross section was observed for 5′-d(A_12_), which is comparable to that of 5′-d(T_12_) and 5′-d(C_12_). In contrast, the cross section for the G-rich sequence, 5′-d(G_12_), is relatively lower. Thus, the observed trend is *σ*_SSB_ (G_12_) < σ_SSB_(C_12_) < *σ*_SSB_(T_12_) ≈ *σ*_SSB_(A_12_). As the incident electron energy of 8.8 eV exceeds the ionization threshold of some nucleobases,^29^ it is expected that the contribution of dissociative ionization, in addition to DEA processes, to the observed cross section values becomes more significant as a possible pathway causing SSBs in the DNA sequences 5′-d(X_12_), where X = A, C, T, G.

### 3.2 Radiosensitization and Low-Energy Electrons (LEEs)

In concomitant chemo-radiation therapy,^15−17,44,45^ the most common radiosensitizing agents currently used in clinics include halogenated nucleosides such as 5-fluorouracil (^5F^U)^14^ and gemcitabine (Gem)^46−48^ and platinum derivatives such as cisplatin.^13,45^ Within concomitant chemo-radiation therapy, radiosensitization combines physicochemical mechanisms^43,50,51^ initiated by secondary LEEs with biological mechanisms, such as the incorporation of metabolites into DNA leading to inhibited cell replication or even apoptosis.^44,52^ The reactivity of ^5F^U,^53−55^ Gem,^56^ and cisplatin^57^ toward LEEs is well-documented in gas-phase studies, although these environments differ from solution-phase conditions encountered in biological settings. Therefore, understanding the mechanisms by which halogenated nucleosides are incorporated into DNA and subsequently increase strand breakage due to LEEs is of the utmost importance. Radiosensitizers activated by LEEs undergo electron-induced reactions, including DEA reactions with secondary LEEs. This process amplifies DNA damage in tumor cells, thus enhancing the efficacy of radiation therapy. This knowledge could lead to the design of novel radiosensitizers explicitly activated by LEEs, potentially improving the efficiency of concomitant chemo-radiation therapy protocols and enhancing the quality of life for cancer patients undergoing radiotherapy.^51,58^ It is noteworthy that other classes of radiosensitizers, including electron-affinity radiosensitizers^59^ such as nimorazole^60^ and tirapazamine^61,62^ as well as pyrimidine-^63−65^ or purine-based analogues,^4,18,66^ are also activated by LEEs.

Rackwitz et al.^18^ demonstrated that the incorporation of 2-fluoroadenine (^2F^A), a component of the chemotherapeutic agent fludarabine,^67−69^ into DNA strands significantly enhances strand breakage upon LEE irradiation. In this study, DNA target strands 5′-d(TT(^2F^AT^2F^A)_3_TT) (sequence a) and 5′-d(TT(ATA)_3_TT) (sequence b) were irradiated at electron energies of 5.5, 10, and 15 eV. Keller et al.^19^ also determined cross sections for DNA single-strand breaks in sequence a. Figure 3C displays the relative number of single-strand breaks in sequences a and b at an incident electron energy of 5.5 eV. It also provides a comparison of cross-section values, which are also listed in Tables S2 and S3. Notably, the cross sections for electron-induced strand breakage show clear energy dependence under the selected electron energies. In the case of the ^2F^A-containing DNA strand, the cross-section value consistently exceeds that of the nonmodified DNA strand. Additionally, both sequences exhibit a higher cross section at 5.5 eV than at compared to 10 or 15 eV. These results indicate that both DNA sequences are more sensitive to 5.5 eV electrons, and the incorporation of two ^2F^A subunits increases, on average, the cross section for strand breakage by a factor of between 1.6 and 1.7 compared to that for nonmodified DNA.

In a follow-up study, Schürmann et al.^4^ investigated the effects of 8-bromoadenine (^8Br^A), a halogenated purine with potential radiosensitizing properties,^70−72^ on electron-induced DNA strand breakages. They irradiated DNA with electrons of energy ranging from 0.5 to 9 eV. As depicted in Figure 3C, the absolute cross sections for strand breakage in both DNA strands, 5′-d(TT(ATA)_3_TT) and 5′-d(TT(^8Br^ATA)_3_)TT), exhibit resonant behavior with a pronounced broad structure at around 7 eV. Within this energy range, the determined cross-section values (summarized in Tables S2 and S3) for the ^8Br^A-modified DNA strand consistently surpass those of the nonmodified sequence. On average, the presence of ^8Br^A increases the cross sections for electron-induced DNA strand breaks by a factor of (1.9 ± 0.6). This comparison highlights the effects induced by ^8Br^A in relation to ^5Br^U, a model for the extensively studied radiosensitizer 5-bromo-2′-deoxyuridine.^59,73−75^

Keller et al.^76^ investigated the sensitization of DNA with ^5Br^U by determining absolute cross sections for electron-induced DNA strand breakage in target sequences 5′-d(TT(XYX)_3_TT), where X = A, C, G and Y = T, and with ^5Br^U upon LEE irradiation with an energy of 18 eV. As presented in Tables S2 and S3, the determined cross sections are strongly influenced by the nucleobase composition. The target strand 5′-d(TT(ATA)_3_TT) displayed the highest cross section while 5′-d(TT(GTG)_3_TT) displayed the lowest according to the trend 5′-d(TT(GTG)_3_TT) < 5′-d(TT(CTC)_3_TT) < 5′-d(TT(ATA)_3_TT). This trend holds even when the central three thymine nucleobases are replaced by ^5Br^U. At 18 eV, the determined cross sections for strand breakage in both nonmodified oligonucleotides and ^5Br^U-modified oligonucleotides are similar. Consequently, the calculated enhancement factors for single-strand breakage yields range from 1.14 for 5′-d(TT(CYC)_3_TT), Y = T, ^5Br^U, to 1.66 for 5′-d(TT(GYG)_3_TT), Y = T, ^5Br^U. Therefore, the damage induced by LEEs with an energy of 18 eV appears to be more sensitive to the nucleobase sequence than to the presence of ^5Br^U.

Additionally, Rackwitz et al.^20^ investigated the sensitization of DNA with ^5F^U, a commercially available radiosensitizer currently used in cancer chemo- and radiotherapy treatments. Through irradiation of two DNA sequences 5′-d(TT(XTX)_3_TT), where X= A, ^5F^U, with LEEs of 10 eV, it was found that the incorporation of ^5F^U enhances the cross section for electron-induced strand breakage by a factor of (1.6 ± 0.5).

In summary, the studies outlined here have provided valuable insights into the mechanisms underlying physicochemical radiosensitization, particularly in the context of LEE interactions with DNA. These findings contribute to a more comprehensive understanding of how radiosensitizers can impact DNA strand breakage, as investigated though AFM imaging of DNA origami nanostructures.

### 3.3 Exploring Higher-Order DNA Structures: G-Quadruplexes

**Figure 4:**
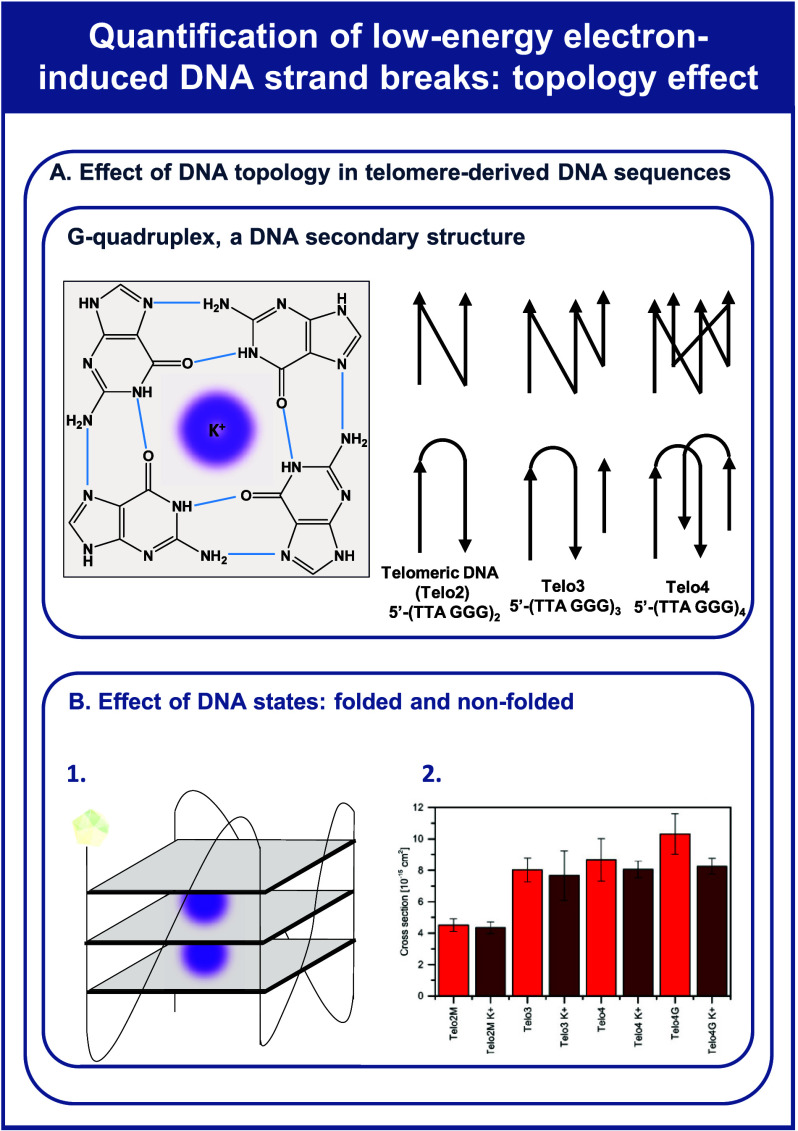
Panel A depicts a planar G-quadruplex alongside telomere-derived DNA sequences. Panel B illustrates the effect of folded and nonfolded DNA states on electron-induced DNA single-strand breaks. It includes (1) possible G-quadruplex conformation and (2) a comparison between the determined absolute cross sections for electron-induced single strand breaks in various telomere-derived DNA sequences, in either their nonfolded (light red) or folded states (dark red) in the presence of K^+^. Panel B was adapted from ref (24).

DNA, in addition to its well-known double-helix structure, can adopt higher-order structures such as G-quadruplexes,^77−79^ as schematically depicted in Figure 4A. They form when two or more G-tetrads come together, often facilitated by metal ions such as K^+^. In a G-quadruplex, four guanine nucleobases are arranged in a planar G-tetrad by eight Hoogsteen hydrogen bonds (represented in blue) and a central cation. This structural motif is prevalent in the human G-rich telomeric sequences, typically consisting of 5′-d(TTAGGG) units. The repetition of these sequences allows for the formation of G-quadruplexes. Figure 4B shows a comparison between absolute cross sections for electron-induced single-strand breaks in telomer-derived DNA sequences in nonfolded and folded states in the presence of K^+^. Significantly, the formation of G-quadruplexes has been observed to protect telomeric DNA against low-energy electrons.

### 3.4 Effect of Oligonucleotide Length on Electron-Induced DNA Strand Breaks

To better understand how oligonucleotide length influences electron-induced DNA strand breaks, Ebel et al.^25^ irradiated polyadenine sequences (poly(A)) with varying electron energies (5.0, 7.0, 8.4, and 10 eV). Figure 5.1 shows the determined absolute cross sections for strand breakage in poly(A) oligonucleotides at different electron energies, with detailed values provided in Tables S2 and S3. The absolute cross sections for strand breakage in poly(A) at different electron energies reveal a consistent energy dependency pattern across all DNA sequences. Specifically, at 8.4 eV, the strand break cross-section trend is as follows: *d*(A_4_) < *d*(A_20_) ≈ *d*(A_8_) < *d*(A_12_) < *d*(A_16_). Figure 5.2 compares the estimated geometrical cross section with the experimentally determined cross sections for each poly(A) sequence. Strikingly, the experimental cross sections are approximately 1 order of magnitude smaller than the estimated geometrical cross sections for each sequence. For 7 and 8.4 eV and sequences shorter than 16 nucleotides, determined cross sections increase quasi-linearly with the oligonucleotide length. Within the electron energy range studied, the cross section for strand breakage in the DNA sequence *d*(A_20_) consistently remains lower than in shorter sequences. An explanation lies in a possible conformational behavior of *d*(A_20_), as depicted in Figure 5.3, suggesting the formation of A-duplexes, potentially leading to an underestimation of strand break cross sections during AFM imaging analysis.

**Figure 5:**
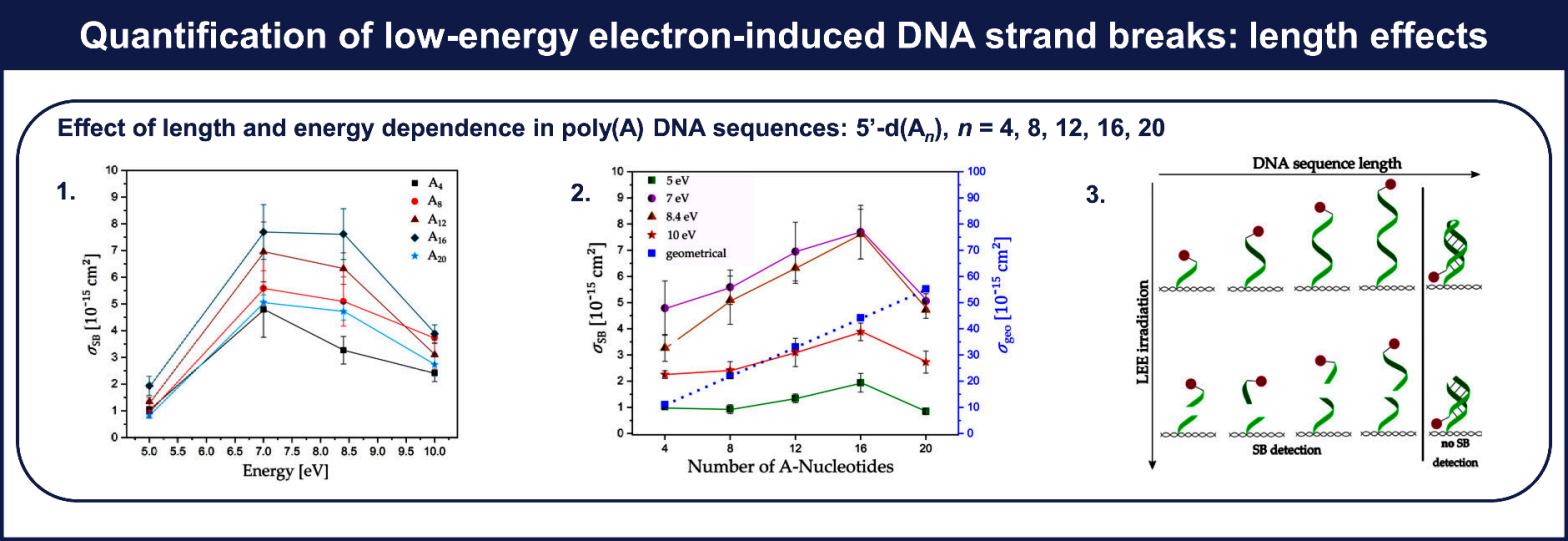
Effects of length and incident electron energy on electron-induced single-strand breaks in poly(A). For 5′-d(A_*n*_), in which *n* = 4, 8, 12, 16, 20, it includes (1) a comparison between experimental absolute cross sections for electron-induced single-strand breaks, σ_SB_, as a function of electron energy, (2) a comparison between σ_SB_ measured at specific electron energies and estimated geometrical cross sections, and (3) a schematic representation of the conformational change of a single-stranded DNA with increasing length and electron-induced strand break. Hydrogen bonding prevents the release of the biotin label (red spot). Adapted from ref (25).

### 3.5 Determining Absolute Cross Sections for Electron-Induced Double Strand Breaks (DSBs)

In previous sections, we discussed how DNA origami nanostructures can accommodate various DNA systems on a single platform to investigate the impact of different qualities of radiation on DNA. This subsection introduces an innovative experimental approach proposed by Ebel et al.,^2^ as illustrated in Figure 6.1, to quantify absolute cross sections for electron-induced double strand breaks (DSBs), *σ*_DSB_.

**Figure 6:**
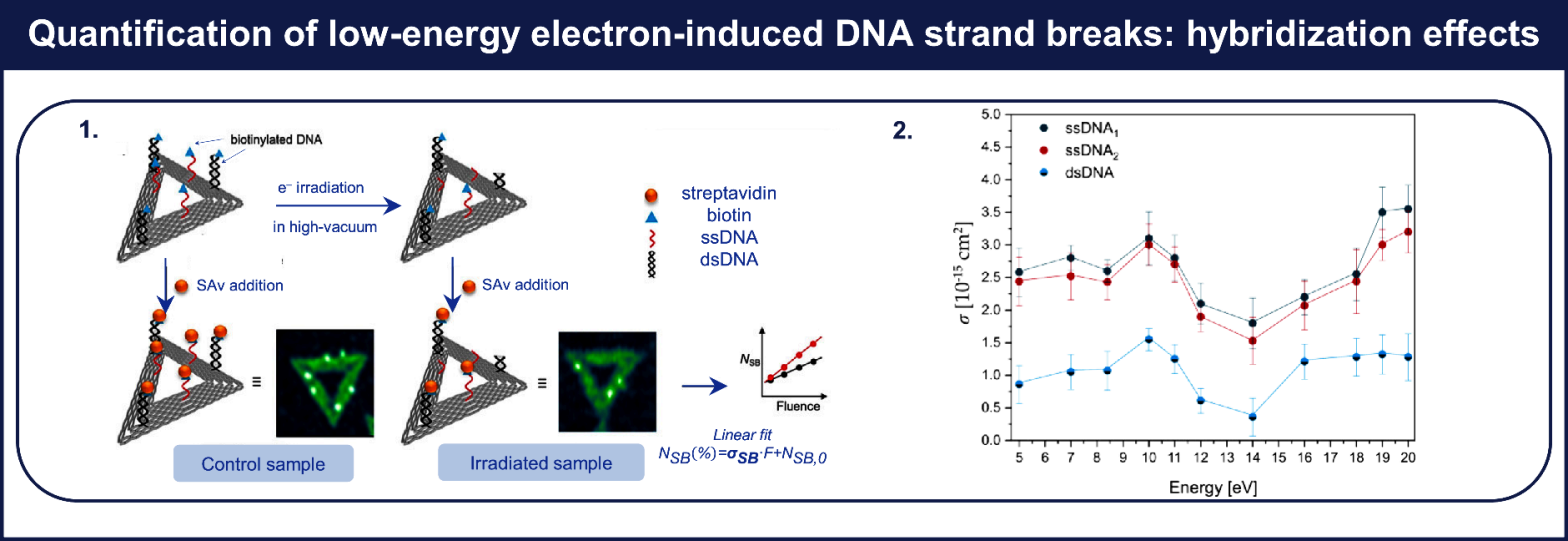
Hybridization effects on electron-induced DNA strand breaks. (1) Experimental scheme for determining absolute cross sections for electron-induced double strand breaks (DSBs). (2) Comparison between absolute cross sections for strand breakage in single-stranded DNA sequences (ssDNA1 and ssDNA2) and in a double-stranded DNA sequence (dsDNA) as a function of the electron energy. Adapted from ref (2).

In the initial step of this approach, Rothemund triangles^23^ serve as platforms for exposing three hairpin structures, composed of the double-stranded DNA (dsDNA) sequence 5′-d((CAC)_4_T(Bt-dT)T_2_(GTG)_4_), held together by a single-stranded biotin-labeled DNA loop 5′-d(T(Bt-dT)T_2_) and three single-stranded DNA sequences (ssDNA). Following irradiation with LEEs under high-vacuum conditions, the remaining intact DNA sequences are labeled with streptadivin (SAv), making them visible in AFM images. To determine the absolute cross sections for electron-induced DSBs, we used a methodology similar to that employed for the determination of absolute cross sections for electron-induced SSBs (as discussed earlier). Figure 6.2 provides a comparison between the experimentally determined cross sections for strand breakage in the dsDNA sequence and in the ssDNA sequences 5′-d((CAC)_4_) and 5′-d((GTG)_4_), referred to as ssDNA_1_ and ssDNA_2_, respectively. Within the electron energy range of 5 to 20 eV, both *σ*_DSB_ in dsDNA and *σ*_SSB_ in ssDNA_1_ and ssDNA_2_ exhibit qualitatively the same response to electron energy. They all display a prominent peak at 10 eV, and an additional maximum at 7 eV is observed only in cross sections for ssDNA_1_ and ssDNA_2_. Overall, the determined yields for SSBs in ssDNA sequences 1 and 2 are about 3 times higher than the yield for DSBs in dsDNA. These observations suggest that the process of DEA predominantly underlies electron-induced DNA single- and double-strand breaks in the lower energy range (<14 eV). At higher electron energies of around 14 to 15 eV, all cross sections reach minima before increasing toward 20 eV. This suggest that different electron-induced reactions, such as dissociative ionization or dipolar dissociations, may begin to contribute to DNA strand breakage at higher electron energies.

## 4 Conclusions

In this Account, we have presented results of our research into DNA radiation damage, employing DNA nanotechnology and atomic force microscopy (AFM). Our investigations have unveiled critical mechanisms underlying radiation-induced DNA damage, ranging from the initiation of DNA strand breaks by VUV photons to the enhanced sensitization of DNA to strand breakage through the incorporation of halogenated nucleobases. We have also explored the vulnerabilities of higher-order DNA structures, such as G-quadruplexes, to electron-induced damage and delved into the effects of oligonucleotide length and nucleobase composition on electron-induced DNA strand breaks. In conclusion, probing DNA radiation damage by DNA nanotechnology has several advantages:

### 4.1 Accurate and Absolute Quantification

By determining absolute cross sections for DNA strand breakage, our technique provides benchmark values which can be compared directly with other experimental and theoretical studies and which can be used to determine quantum yields for strand breakages.

### 4.2 Control and Functionalization

As the DNA sequences to be studied are artificially synthesized, they can be freely chosen, and accurate sequence-dependencies can be revealed.

### 4.3 Comparison

This approach enables the simultaneous irradiation of two target oligonucleotide sequences within a single experiment, allowing efficient comparison across various DNA systems, for example, to determine accurate enhancement factors for radiosensitizers.

### 4.4 Single-Molecule Sensitivity and Cost Effectiveness

AFM imaging allows for the detection and analysis of DNA strand breaks at the single-molecule level, reducing the amount of DNA required for studies.

Our approach based on DNA nanotechnology and AFM analysis already provided detailed and invaluable insights into radiation damage to DNA. While the application of DNA nanotechnology remains in its early stages, it is advancing rapidly and with an immense potential to open new avenues in the field of DNA damage research. Novel DNA origami structures can be tailored to study the effect of various radiation qualities, probe higher-order DNA-like i-motifs, or even examine the effect of emerging cancer therapeutics or other molecular systems pertinent for nanomedicine and biomedical applications. Investigations on mechanisms and processes for radiation damage can be extended to other innovative cancer treatment approaches, also including photodynamic therapy.^29^

Furthermore, the potential applications of chemically modified DNA origami-based approaches are vast, encompassing targeted drug delivery, nanoparticle-based radiosensitization, and DNA damage sensing. It is worth noting, however, that DNA origami nanostructures are sensitive to various experimental conditions, including changes in temperature, pressure, pH, salt concentration, and exposure to ionizing radiation. Therefore, ensuring the structural integrity and durability remains key to open potential clinical applications.

## Supporting Information Available

Overviews of σ_SSB_ values for single-strand breakage upon VUV irradiation and σ_SSB_ for low-energy electron-induced single-strand breakage (0.5 to 8.4 and 8.8 to 18 eV) (PDF)
