# Supplementary material for: Unraveling the Complexity of DNA Radiation Damage Using DNA Nanotechnology
Source: Acc Chem Res. 2024 May 23;57(11):1608–19. doi: 10.1021/acs.accounts.4c00121 (PMC11154965; doi:10.1021/acs.accounts.4c00121)
Supplement: Supplementary file 1 — ar4c00121_si_001.pdf [file ar4c00121_si_001.pdf]

# Supporting Information

# Unraveling the complexity of DNA radiation damage using DNA nanotechnology

João Ameixa[1,2], Ilko Bald[1*]

[1]Institute of Chemistry, Hybrid Nanostructures, University of Potsdam, Karl-Liebknecht-Str. 24-25, 14476, Potsdam, Germany, Email: bald@uni-potsdam.de
[2]Centre of Physics and Technological Research (CEFITEC), Department of Physics, NOVA School of Science and Technology, University NOVA of Lisbon, Campus de Caparica, 2829-516, Portugal

Table S1 - Overview of absolute cross sections ($\sigma_{SSB}$) values for single-strand breakage for the different DNA sequences upon VUV irradiation.

**Vacuum-ultraviolet photons**

| **Energy (eV)** | **DNA sequence** | **$\sigma_{SSB}$ ($x10^{-16}$ $cm^2$)** | **Reference** |
|---|---|---|---|
| 6.50 | 5’-d(TT(CTC)$_3$TT) | 0.9±0.4 | [1] |
| | 5’-d(TT(ATA)$_3$TT) | 1.2±0.3 | [1] |
| 7.29 | 5’-d(TT(CTC)$_3$TT) | 0.6±0.1 | [1] |
| | 5’-d(TT(ATA)$_3$TT) | 0.5±01 | [1] |
| 8.44 | 5’-d(G$_{12}$) | 2.3±0.2 | [2] |
| | 5’-d(TT(G$^{5Br}$UG)$_3$TT) | 7.9±0.4 | [3] |
| | 5’-d(C$_{12}$) | 1.7±0.1 | [2] |
| | 5’-d(TT(C$^{5Br}$UC)$_3$TT) | 10.2±1.1 | [3] |
| | 5’-d(T$_{12}$) | 2.1±0.2 | [2] |
| | 5’-d(TT(T$^{5Br}$UT)$_3$TT) | 10.5±1.3 | [3] |
| | 5’-d(TTG$^{5Br}$UTT) | 3.9±1.2 | [3] |
| | 5’-d(TTGA$^{5Br}$UTT) | 3.5±1.3 | [3] |
| | 5’-d(TTGAA$^{5Br}$UTT) | 3.4±0.8 | [3] |
| | 5’-d(TTGAAA$^{5Br}$UTT) | 3.0±0.5 | [3] |
| | 5’-d(TTGAAAA$^{5Br}$UTT) | 3.1±0.7 | [3] |
| | 5’-d(TTGAAAAA$^{5Br}$UTT) | 2.5±0.3 | [3] |
| | 5’-d(A$_{12}$) | 2.1±0.3 | [2] |
| | 5’-d(TT(CTC)$_3$TT) | 2.2±0.4 | [1] |
| | 5’-d(TT(ATA)$_3$TT) | 2.8±0.2 | [1] |
| | 5’-d(TT(ATA)$_3$TT) | 2.2±0.7 | [3] |
| | 5’-d(TT($^{8Br}$ATA)$_3$TT) | 2.7±0.6 | [3] |
| | 5’-d(TT(ATA)$_3$TT) | 4.1±0.6* | [3] |
| | 5’-d(TT($^{8Br}$ATA)$_3$TT) | 3.9±0.4* | [3] |
| 8.94 | 5’-d(TT(CTC)$_3$TT) | 4.9±0.5 | [1] |
| | 5’-d(TT(ATA)$_3$TT) | 6.9±1.1 | [1] |

$^{5Br}$U - 5-bromouracil; $^{8Br}$A – 8-bromoadenine

*Irradiation on Si as substrate material.

Table S2 - Overview of the absolute cross sections ($\sigma_{SSB}$) for low-energy electron-induced single-strand breakage for different DNA sequences in the energy range from 0.5 to 8.4 eV.

**Low-energy electrons (LEEs)**

| **Energy (eV)** | **DNA sequence** | **$\sigma_{SSB}$ ($\times 10^{-15}$ cm$^2$)** | **References** |
|---|---|---|---|
| 0.5 | 5'-d(TT(ATA)$_3$TT) | 2.9±0.6 | 4 |
| 1 | 5'-d(TT(ATA)$_3$TT) | 3.5±1.7 | 4 |
| 2 | 5'-d(TT(ATA)$_3$TT) | 5.9±0.3 | 4 |
| 5 | 5'-d(A$_4$) | 1.03±0.12 | 5 |
| | 5'-d(A$_8$) | 0.93±0.17 | 5 |
| | 5'-d(A$_{12}$) | 1.34±0.17 | 5 |
| | 5'-d(A$_{16}$) | 1.94±0.36 | 5 |
| | 5'-d(A$_{20}$) | 0.85±0.13 | 5 |
| 5.5 | 5'-d(TT($^{5F}$UT$^{5F}$U)$_3$TT) | 11.7±1.1 | 6 |
| | 5'-d(TT(ATA)$_3$TT) | 13.6±0.2 | 7 |
| | 5'-d(TT($^{2F}$AT$^{2F}$ A)$_3$TT) | 21.2±0.9 | 7 |
| 7 | 5'-d(A$_4$) | 4.79±1.04 | 7 |
| | 5'-d(A$_8$) | 5.59±0.66 | 7 |
| | 5'-d(A$_{12}$) | 6.95±1.12 | 7 |
| | 5'-d(A$_{16}$) | 7.69±1.02 | 7 |
| | 5'-d(A$_{20}$) | 5.06±0.28 | 7 |
| | 5'-d(TT(ATA)$_3$TT) | 11±3 | 4 |
| 8.4 | 5'-d(A$_4$) | 3.27±0.51 | 5 |
| | 5'-d(A$_8$) | 5.09±0.92 | 5 |
| | 5'-d(A$_{12}$) | 6.32±0.59 | 5 |
| | 5'-d(A$_{16}$) | 7.62±0.95 | 5 |
| | 5'-d(A$_{20}$) | 4.72±0.32 | 5 |

$^{2F}$A – 2-fluoroadenine; $^{8Br}$A – 8-bromoadenine; $^{5F}$U- 5-fluorouracil; $^{5Br}$U – 5-bromouracil

Table S3. Overview of the absolute cross sections ($\sigma_{SSB}$) for low-energy electron-induced single-strand breakage for different DNA sequences in the energy range from 8.8 to 18 eV.

**Low-energy electrons (LEEs)**

| **Energy (eV)** | **DNA sequence** | **$\sigma_{SSB}$ ($x10^{-15}$ $cm^2$)** | **References** |
|---|---|---|---|
| 8.8 | 5'-d($A_{12}$) | 3.9±0.4 | 2 |
| | 5'-d($C_{12}$) | 3.4±0.5 | 2 |
| | 5'-d($G_{12}$) | 3.1±0.2 | 2 |
| | 5'-d($T_{12}$) | 3.7±0.2 | 2 |
| | 5'-d(TT(GGGATT)$_2$T) | 6.78±0.54 | 8 |
| | 5'-d(TT(GGGGTT)$_2$T) | 6.02±0.31 | 8 |
| | 5'-d(TT(GGGATT)$_3$T) | 8.02±0.76 | 8 |
| | 5'-d(TT(GGGATT)$_4$T) | 8.67±1.35 | 8 |
| | 5'-d(TT(GGGGTT)$_4$T) | 10.30±1.29 | 8 |
| | 5'-d(TT(TTAGGG)$_2$T) | 6.74±0.56 | 8 |
| | 5'-d(TT(TGTGTGA)$_2$T) | 4.51±0.27 | 8 |
| | 5'-d(TT(GGGATT)$_4$T) | 8.05±0.5† | 8 |
| | 5'-d(TT(GGGGTT)$_4$T) | 8.26±0.50† | 8 |
| | 5'-d(TT(GGGATT)$_3$T) | 7.66±1.57† | 8 |
| | 5'-d(TT(TGTGTGA)$_2$T) | 4.35±0.38† | 8 |
| 9 | 5'-d(TT(ATA)$_3$TT) | 9.1±0.9 | 4 |
| 10 | 5'-d($A_4$) | 2.25±0.15 | 5 |
| | 5'-d($A_8$) | 2.42±0.33 | 5 |
| | 5'-d($A_{12}$) | 3.10±0.54 | 5 |
| | 5'-d($T_{12}$) | 7.9±0.7 | 6 |
| | 5'-d($A_{16}$) | 3.88±0.34 | 5 |
| | 5'-d($A_{20}$) | 2.73±0.42 | 5 |
| | 5'-d(TT(ATA)$_3$TT) | 8.0±1.2 | 7 |
| | 5'-d(TT($^{2F}$AT$^{2F}$ A)$_3$TT) | 13.4±2.2 | 7 |
| | 5'-d(TT-(GTG)$_3$TT) | 5.7±1.2 | 7 |
| | 5'-d(TT($^{5F}$UT$^{5F}$U)$_3$TT) | 13.1±2.5 | 6 |
| | 5'-d(TT(A$^{5F}$UA)$_3$TT) | 12.0±2.0 | 6 |
| 15 | 5'-d(TT(ATA)$_3$TT) | 3.9±0.9 | 7 |
| | 5'-d(TT($^{2F}$AT$^{2F}$ A)$_3$TT) | 6.7±1.9 | 7 |
| 18 | 5'-d(TT(ATA)$_3$TT) | 60±8.6 | 9 |
| | 5'-d(TT(CTC)$_3$TT) | 26.6±9.3 | 9 |
| | 5'-d(TT(GTG)$_3$TT) | 22.1±8.7 | 9 |
| | 5'-d(TT(A$^{5Br}$UA)$_3$TT) | 70.6±2.4 | 9 |
| | 5'-d(TT(C$^{5Br}$UC)$_3$TT) | 30.4±8.5 | 9 |
| | 5'-d(TT(G$^{5Br}$UG)$_3$TT) | 36.7±9.0 | 9 |

$^{2F}$A – 2-fluoroadenine; $^{8Br}$A – 8-bromoadenine; $^{5F}$U- 5-fluorouracil; $^{5Br}$U – 5-bromouracil

†Studies performed in the presence of 100 mM KCl.

## References


(1) Vogel, S.; Rackwitz, J.; Schürman, R.; Prinz, J.; Milosavljević, A. R.; Réfrégiers, M.; Giuliani, A.; Bald, I. Using DNA Origami Nanostructures to Determine Absolute Cross Sections for UV Photon-Induced DNA Strand Breakage. *Journal of Physical Chemistry Letters* **2015**, *6* (22), 4589–4593. https://doi.org/10.1021/acs.jpclett.5b02238.

(2) Vogel, S.; Ebel, K.; Schürmann, R. M.; Heck, C.; Meiling, T.; Milosavljevic, A. R.; Giuliani, A.; Bald, I. Vacuum-UV and Low-Energy Electron-Induced DNA Strand Breaks – Influence of the DNA Sequence and Substrate. *ChemPhysChem* **2019**, *20* (6), 823–830. https://doi.org/10.1002/CPHC.201801152.

(3) Vogel, S.; Ebel, K.; Heck, C.; Schürmann, R. M.; Milosavljević, A. R.; Giuliani, A.; Bald, I. Vacuum-UV Induced DNA Strand Breaks-Influence of the Radiosensitizers 5-Bromouracil and 8-Bromoadenine. *Physical Chemistry Chemical Physics* **2019**, *21* (4), 1972–1979. https://doi.org/10.1039/c8cp06813e.

(4) Schürmann, R.; Tsering, T.; Tanzer, K.; Denifl, S.; Kumar, S. V. K.; Bald, I. Resonant Formation of Strand Breaks in Sensitized Oligonucleotides Induced by Low-Energy Electrons (0.5–9 EV). *Angewandte Chemie - International Edition* **2017**, *56* (36), 10952–10955. https://doi.org/10.1002/anie.201705504.

(5) Ebel, K.; Bald, I. Length and Energy Dependence of Low-Energy Electron-Induced Strand Breaks in Poly(A) DNA. *Int J Mol Sci* **2020**, *21* (1), 111. https://doi.org/10.3390/ijms21010111.

(6) Rackwitz, J.; Ranković, M. L.; Milosavljević, A. R.; Bald, I. A Novel Setup for the Determination of Absolute Cross Sections for Low-Energy Electron Induced Strand Breaks in Oligonucleotides – The Effect of the Radiosensitizer 5-Fluorouracil. *European Physical Journal D* **2017**, *71* (2), 1–9. https://doi.org/10.1140/epjd/e2016-70608-4.

(7) Rackwitz, J.; Kopyra, J.; Dąbkowska, I.; Ebel, K.; Ranković, M. Lj.; Milosavljević, A. R.; Bald, I. Sensitizing DNA Towards Low-Energy Electrons with 2-Fluoroadenine. *Angewandte Chemie - International Edition* **2016**, *55* (35), 10248–10252. https://doi.org/10.1002/anie.201603464.

(8) Rackwitz, J.; Bald, I. Low-Energy Electron-Induced Strand Breaks in Telomere-Derived DNA Sequences—Influence of DNA Sequence and Topology. *Chemistry – A European Journal* **2018**, *24* (18), 4680–4688. https://doi.org/10.1002/CHEM.201705889.

(9) Keller, A.; Rackwitz, J.; Cauët, E.; Liévin, J.; Körzdörfer, T.; Rotaru, A.; Gothelf, K. V.; Besenbacher, F.; Bald, I. Sequence Dependence of Electron-Induced DNA Strand Breakage Revealed by DNA Nanoarrays. *Sci Rep* **2014**, *4* (1), 1–6. https://doi.org/10.1038/srep07391.
